# Melanoma skin cancer statistics derived from 7442 Japanese patients: Japanese melanoma study

**DOI:** 10.1007/s10147-025-02747-9

**Published:** 2025-04-07

**Authors:** Yasuhiro Fujisawa, Shusuke Yoshikawa, Tatsuya Takenouchi, Shoichiro Mori, Jun Asai, Hisashi Uhara, Yuki Ichigosaki, Taku Fujimura, Yoshiyuki Nakamura, Yasuhiro Nakamura, Fumitaka Ohno, Takeshi Fukumoto, Toshiyuki Ozawa, Kenjiro Namikawa, Satoru Sugihara, Toshihiko Hoashi, Takatoshi Shimauchi, Yu Sawada, Hiroaki Iwata, Taku Maeda, Takuya Miyagawa, Yoshitsugu Shibayama, Naohito Hatta, Akiko Kishi, Masashi Ishikawa, Hisao Kawahira, Norito Katoh, Ryuhei Okuyama

**Affiliations:** 1 Department of Dermatology, Ehime University, 454 Shizugawa, Toon, Ehime 791-0204 Japan; 2 Department of Dermatology, Shizuoka Cancer Center, Nagaizumi, Japan; 3 Division of Dermatology, Niigata Cancer Center Hospital, Niigata, Japan; 4 Department of Dermatology, Nagoya University, Nagoya, Japan; 5 Department of Dermatology, Kyoto Prefectural University of Medicine Graduate School of Medical Science, Kyoto, Japan; 6 Department of Dermatology, Sapporo Medical University, Sapporo, Japan; 7 Department of Dermatology and Plastic Surgery, Kumamoto University, Kumamoto, Japan; 8 Department of Dermatology, Tohoku University Graduate School of Medicine, Sendai, Japan; 9 Department of Dermatology, Institute of Medicine, University of Tsukuba, Tsukuba, Japan; 10 Department of Skin Oncology/Dermatology, Saitama Medical University International Medical Center, Hidaka, Japan; 11 Department of Dermatology, Graduate School of Medical Sciences, Kyushu University, Fukuoka, Japan; 12 Division of Dermatology, Department of Internal Related, Graduate School of Medicine, Kobe University, Kobe, Japan; 13 Pharmaco-Physiology & Kinetics Collaborative Research Division, Graduate School of Medicine, Osaka Metropolitan University, Osaka, Japan; 14 Department of Dermatologic Oncology, National Cancer Center Hospital, Tokyo, Japan; 15 Department of Dermatology, Okayama University, Okayama, Japan; 16 Department of Dermatology, Nippon Medical School, Bunkyo, Japan; 17 Department of Dermatology, Hamamatsu University School of Medicine, Hamamatsu, Japan; 18 Department of Dermatology, University of Occupational and Environmental Health, Hakata, Japan; 19 Department of Dermatology, Gifu University Graduate School of Medicine, Gifu, Japan; 20 Department of Plastic and Reconstructive Surgery, Hokkaido University Hospital, Sapporo, Japan; 21 Department of Dermatology, University of Tokyo Graduate School of Medicine, Tokyo, Japan; 22 Department of Dermatology, Faculty of Medicine, Fukuoka University, Fukuoka, Japan; 23 Department of Dermatology, Toyama Prefectural Central Hospital, Toyama, Japan; 24 Department of Dermatology, Toranomon Hospital, Tokyo, Japan; 25 Department of Dermatology, Saitama Cancer Center Hospital, Ina, Japan; 26 Department of Dermatology, Kagoshima University Graduate School of Medical and Dental Sciences, Kagoshima, Japan; 27 Department of Dermatology, Shinshu University, Matsumoto, Japan

**Keywords:** Malignant melanoma, Statistics, Clinical subtype, Survival

## Abstract

**Background:**

Malignant melanoma (MM) is a rare but aggressive cutaneous cancer, accounting for only 2% of skin cancers in Japan but nearly half of skin cancer-related deaths. While the global incidence of MM is rising, its epidemiology varies significantly by ethnicity and geographic region. In Japan, melanoma incidence remains lower than in Western countries, with acral lentiginous melanoma (ALM) being the most prevalent subtype. However, comprehensive epidemiological and clinical data remain limited.

**Methods:**

We analyzed data from 7442 Japanese melanoma patients collected between 2005 and 2022 through the Japanese Melanoma Study (JMS). Demographic, clinical, and survival data were evaluated, including subtype distribution, TNM staging, and treatment outcomes.

**Results:**

ALM was the most common subtype (40.8%), followed by superficial spreading melanoma (20.2%). Lymph node metastasis was observed in 28.6% of cases, and distant metastasis in 10.9%. The BRAF mutation rate was 27.2%, with significantly lower frequencies in ALM (8.5%) and mucosal melanoma (4.8%). Among Stage IV patients, those treated with both immune checkpoint inhibitors (ICIs) and BRAF(+ MEK) inhibitors demonstrated significantly improved survival compared to chemotherapy alone (*P* < 0.05). Adjuvant BRAF(+ MEK) inhibitor therapy also resulted in superior relapse-free survival compared to those who did not receive adjuvant therapy (*P* < 0.005).

**Conclusion:**

This study provides the largest dataset of Japanese melanoma patients to date, highlighting distinct epidemiological and clinical characteristics. Given their lower BRAF mutation rates and the limited efficacy of current ICI treatments, these findings emphasize the urgent need for optimize immunotherapy strategies in Japanese melanoma patients.

## Introduction

Malignant melanoma (MM) arises from the pigment cells of the skin and is a rare form of cutaneous cancer. Although the annual incidence of melanoma in Japan has been reported to be 1–2 patients/100,000 [1, 2], MM-related mortalities are close to half the cutaneous cancer deaths because of its aggressiveness and high rates of metastases [3].

The epidemiology of melanoma is highly heterogeneous across different ethnic groups and geographic regions, influenced by genetic, environmental, and cultural factors. The variations are so diverse that it becomes urgent to conduct race-specific studies to analyze the disease dynamics and treatment more precisely.

In Japan, the incidence of melanoma is lower than in Western countries [1, 2]. Notably, Japanese melanoma patients exhibit distinct clinical features, with ALM being the most common subtype, accounting for nearly half of all cases [4], a stark contrast to its rarity in Western populations. These unique characteristics underscore the importance of investigating the epidemiology and clinical outcomes of melanoma specifically in the Japanese population.

Recent advances in treatment options, including immune checkpoint inhibitors (ICIs) and BRAF and MEK inhibitors (BRAFi + MEKi), have contributed to a decline in melanoma-related mortality [5, 6]. However, melanoma remains a challenging disease to manage [7]. For Japanese patients, understanding the demographic trends, clinical subtypes, and survival outcomes is critical for developing tailored treatment strategies. However, comprehensive, long-term epidemiological data on melanoma in Japan remain limited.

In 2019, we published the results of this study, which included data on 4,594 Japanese melanoma patients collected from 2005 to 2017 [4]. The present study analyzed an expanded dataset comprising 6790 patients collected from 2005 to 2022. By analyzing an expanded dataset comprising 6790 melanoma cases, we aimed to identify trends in clinical subtypes, treatment responses, and survival outcomes, thereby addressing gaps in the understanding of melanoma in the Japanese population.

## Materials and methods

The Committee of Statistical Survey of Skin Cancer Prognosis of the Japanese Skin Cancer Society started the Japanese Melanoma Study (JMS) to collect data on melanoma patients admitted to 27 institutions across Japan. In 2023, the JMS was expanded to include additional participating institutions and was integrated into a newly established registry study, the Japanese Skin Cancer Registry System (JSCaRS). In the present study, data from the JMS, collected between 2005 and 2022, were utilized for further analysis. The JMS employs an online patient information submission system, which collaborating researchers access at least annually to submit new patient data and update prognostic information, including details of additional treatments administered following any recurrence.

Patient information included age, sex, site of the primary tumor, clinical type, Breslow thickness, Clark level, regression, in-transit or satellite metastases, TNM status as per the American Joint Committee on Cancer (AJCC) 7th Edition (for patients enrolled from 2005 to 2017) or the AJCC 8th Edition (for patients enrolled from 2018 to 2022), type of treatment, and follow-up information such as recurrence type and prognosis. All data regarding vital status and date of death were based solely on investigator reports.

Patient follow-up was conducted at the discretion of each attending physician, generally following the guidelines published by the Japanese Dermatology Association. The 2015 guidelines recommended follow-up intervals as follows: at least every 3 months during the first year and every 6 months for years 2–5 for Stage I disease; every 3 months during the first 3 years and every 6 months for years 4–5 for Stage II disease; and monthly during the first 3 years and every 3 months for years 4–5 for Stage III or higher disease. The guideline was updated in 2019 [8], which recommend follow-up every 6–12 months for years 2–5 for Stage I and IIA disease, and every 3–6 months during the first 2 years, followed by every 6 months for years 3–5 for Stage IIB or higher disease. For elderly patients, follow-up intervals may be extended beyond these recommendations based on clinical judgment.

Survival curves were estimated using the Kaplan–Meier method. A Cox proportional hazards model was employed to calculate the hazard ratio (HR), p-value, and 95% confidence interval (95% CI) for each variable. All statistical analyses were conducted using the open-source statistical software R version 4.2.1, and a p-value of less than 0.05 was considered statistically significant. This manuscript was edited for English language clarity using ChatGPT-4.

This study was approved by the institutional review boards of all participating institutions and the Japanese Dermatology Association. This study was funded by the Japanese Skin Cancer Society.

## Results

### Background data

We analyzed data from 7442 Japanese melanoma patients, including 5177 patients collected between 2005 and 2017 and 2265 patients collected between 2018 and 2022. Among the patients, 3432 were male (46.1%) and 4007 were female, indicating a slight female predominance. The average age at diagnosis was 65.2 years, with a median age of 68.0 years. The average time from tumor notification to the first medical visit was 67.1 months, with a median duration of 24.0 months.

### Primary tumor status

The primary tumor status is summarized in Table 1. As shown in the table, the most common site of the primary tumor was the lower extremity (41.1%), followed by the upper extremity (16.6%), head and neck (14.7%), trunk (14.4%), and mucosa (9.5%). Regarding clinical subtypes, acral lentiginous melanoma (ALM) was the most prevalent (40.8%), followed by superficial spreading melanoma (SSM, 20.2%), nodular melanoma (NM, 10.5%), mucosal melanoma (MCM, 9.5%), lentigo maligna melanoma (LMM, 8.5%), uveal (1.0%), primary unknown (2.1%), and others/not stated (7.5%).

**Table 1:** Primary tumor status of the entire cohort

Tumor site		
	*N*	%
Head/neck	1097	14.7
Trunk	1069	14.4
Upper extremity	1234	16.6
Arm	346	4.6
Hand/finger	490	6.6
Fingernail	398	5.3
Lower extremity	3057	41.1
Leg	626	8.4
Foot/toe	2190	29.4
Toenail	241	3.2
Mucosal	709	9.5
Ocular	75	1.0
Primary unknown	163	2.2
Others/not specified	38	0.5
Total	7442	

Clinical subtype		
	*N*	%
ALM	3038	40.8
SSM	1501	20.2
NM	779	10.5
LMM	629	8.5
MCM	709	9.5
Ocular	75	1.0
Primary unknown	156	2.1
Others/not specified	555	7.5
Total	7442	

*ALM* acral lentiginous melanoma, *SSM* superficial spreading melanoma, *NM* nodular melanoma, *MCM* mucosal melanoma

The average tumor thickness among cutaneous melanoma subtypes (ALM, SSM, NM, and LMM) was 3.90 mm, with a median thickness of 2.20 mm. Ulceration was observed in 27.9% of cases. BRAF mutation status was available for 1,158 patients across all clinical subtypes, with 27.2% found to harbor a BRAF mutation. The frequency of BRAF mutations for each clinical subtype was as follows: ALM, 8.5%; SSM, 60.3%; NM, 46.8%; LMM, 26.9%; MCM, 4.8%; uveal melanoma, 0%; primary unknown, 25.5%; and others/unknown, 26.9%.

### Lymph node status

Overall, pathologically or clinically, lymph node metastasis was identified in 2,132 patients (28.6% of the entire cohort). Among these patients, 390 were also presented with distant metastasis. The frequency of lymph node metastasis for each clinical subtype was as follows: acral lentiginous melanoma (ALM), 23.0%; superficial spreading melanoma (SSM), 33.0%; nodular melanoma (NM), 53.0%; lentigo maligna melanoma (LMM), 9.2%; and mucosal melanoma (MCM), 27.9%.

Sentinel lymph node biopsy was performed on 3748 patients, of whom 969 (31.4%) tested positive for tumor involvement in the sentinel lymph nodes. In-transit metastasis was observed in 476 patients (6.4% of the entire cohort).

### Distant metastasis

Distant metastasis was found in 808 patients representing 10.9% of the entire cohort at the time of diagnosis. The frequency of distant metastasis for each clinical subtype was as follows: ALM, 4.7%; SSM, 5.2%; NM, 11.7%; LMM, 1.7%; MCM, 32.3%; and uveal melanoma, 58.7%.

### TNM staging by AJCC 7th classification of the cutaneous melanoma and survival

A total of 5117 patients participated in the study conducted between 2005 and 2017. Among them, 4103 were diagnosed with cutaneous melanoma, and pathological staging was available for 3892 patients. The distribution of each TNM stage, classified according to the AJCC 7th edition system, is summarized in Table 2. For the T classification, the most common category was Tis (22.7%), followed by T4b (20.2%). Nearly a quarter of the patients (26.0%) had lymph node metastasis, while 5.5% presented distant metastasis at the time of diagnosis.

**Table 2:** TNM staging by AJCC 7th classification of the 3892 cutaneous melanomas

T	No	%
Tis	856	22.0
T1a	595	15.3
T1b	103	2.6
T2a	389	10.0
T2b	132	3.4
T3a	345	8.9
T3b	355	9.1
T4a	300	7.7
T4b	774	19.9
N.S	43	1.1

*N*	No	%
N0	2820	72.5
N1a	292	7.5
N1b	90	2.3
N2a	181	4.7
N2b	90	2.3
N2c	46	1.2
N3	316	8.1
N.S	57	1.5

Stage	No	%
0	846	21.7
IA	559	14.4
IB	405	10.4
IIA	325	8.4
IIB	353	9.1
IIC	304	7.8
IIIA	201	5.2
IIIB	355	9.1
IIIC	324	8.3
IV	219	5.6

*N.S.* not stated, *5Y-DSS* 5-year disease-specific survival, *10Y* 10-year

**Fig. 1:**
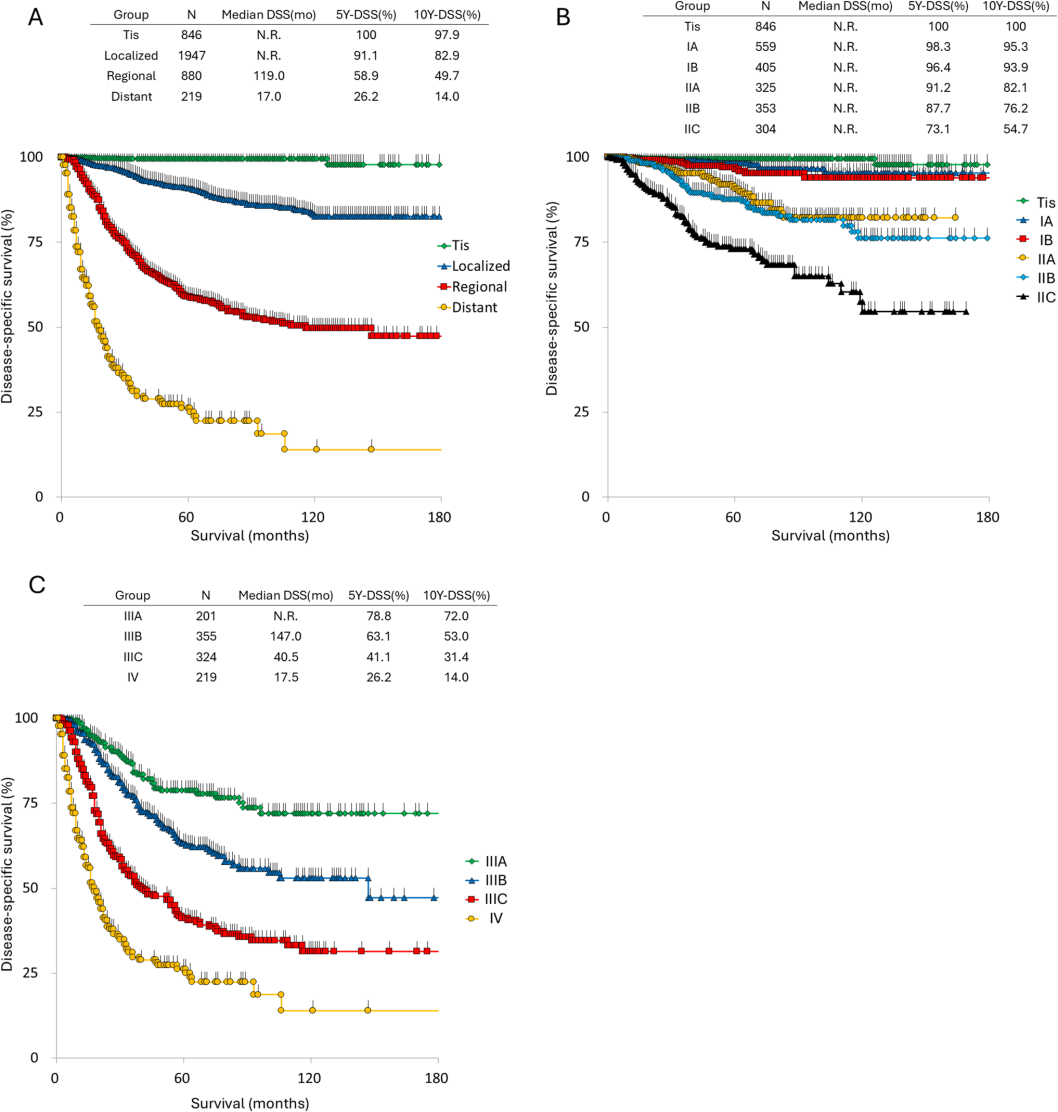
Survival curves of patients enrolled between 2005 and 2017. **A** Survival curves stratified by disease stage: Tis, localized, regional, and distant disease. **B** Survival curves of patients with stages Tis to IIC according to the AJCC 7th classification system. **C** Survival curves of patients with Stages IIIA to IV according to the AJCC 7th classification system. 5Y-DSS: 5-year disease-specific survival, 10Y-DSS: 10-year disease-specific survival, N.R.: not reached

The survival curves generated using the Kaplan–Meier method and 5-year and 10-year disease-specific survival (DSS) rates are shown in Fig. 1. In most stages, DSS did not plateau within 5 years but continued to decline until 10 years. In Stages IIC, IIIB, IIIC, and IV, the rate of survival decline was greater within the first 5 years compared to the 5-to-10-year period. In contrast, in Stages IA, IB, IIA, IIB, and IIIA, the rate of survival decline was greater during the 5-to-10-year period, indicating that patients with earlier stages were more likely to die of the disease after 5 years. These findings underscore the importance of monitoring patients over an entire 10-year period.

### TNM staging by AJCC 8th classification of the cutaneous melanoma and survival

**Fig. 2:**
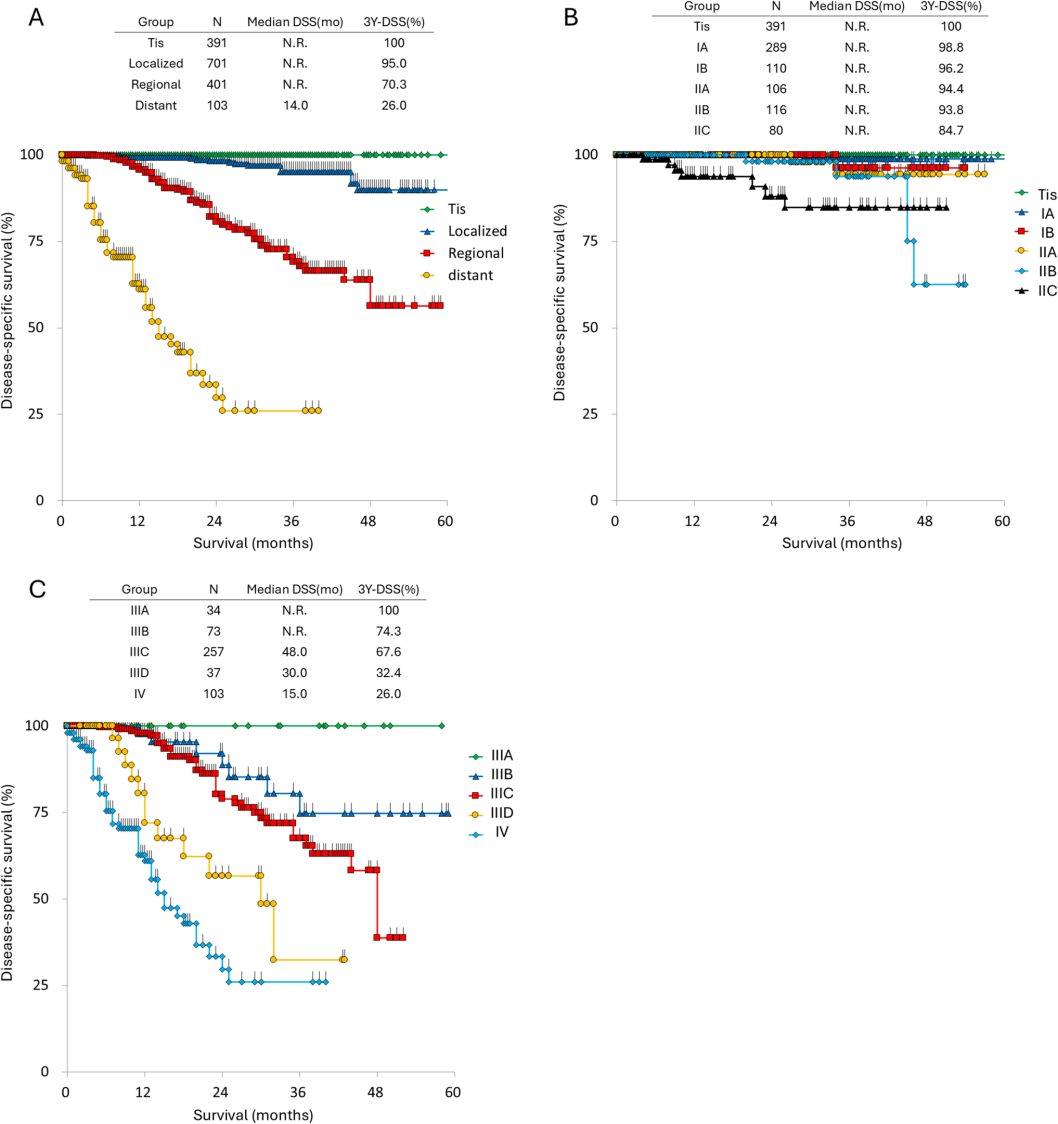
Survival curves of patients enrolled between 2018 and 2022. **A** Survival curves stratified by disease stage: Tis, localized, regional, and distant disease. **B** Survival curves of patients with stages Tis to IIC according to the AJCC 8th classification system. Survival curves of patients with Stages IIIA to IV according to the AJCC 8th classification system. 3Y-DSS: 3-year disease-specific survival, N.R.: not reached

A total of 2265 cases were collected between 2018 and 2022. Among these, 1844 patients were diagnosed with cutaneous melanoma, and pathological staging was available for 1596 patients. The distribution of each TNM stage, classified according to the AJCC 8th edition system, is summarized in Table 3. Although the overall trend remained consistent, the proportion of patients diagnosed at an early stage was higher during the 2018–2022 period compared to the 2005–2017 period. Survival curves generated using the Kaplan–Meier method and the 3-year DSS rates are presented in Fig. 2. However, as the follow-up period is still immature compared to the 2005–2017 dataset, drawing definitive conclusions from this cohort remains challenging.

**Table 3:** TNM staging by AJCC 8th classification of the 1596 cutaneous melanomas

T	No	%
Tis	392	24.6
T0	6	0.4
T1a	231	14.5
T1b	82	5.1
T2a	143	9.0
T2b	50	3.1
T3a	136	8.5
T3b	124	7.8
T4a	120	7.5
T4b	293	18.4
N.S	19	1.2

*N*	No	%
N0	1,101	69.0
N1a	178	11.2
N1b	31	1.9
N1c	17	1.1
N2a	75	4.7
N2b	40	2.5
N2c	17	1.1
N3a	15	0.9
N3b	46	2.9
N3c	46	2.9
N.S	30	1.9

Stage	No	%
0	391	24.5
IA	289	18.1
IB	110	6.9
IIA	106	6.6
IIB	116	7.3
IIC	80	5.0
IIIA	34	2.1
IIIB	73	4.6
IIIC	257	16.1
IIID	37	2.3
IV	103	6.5

*N.S* not stated, *3Y-DSS* 3-year disease-specific survival

### Mucosal melanoma

**Fig. 3:**
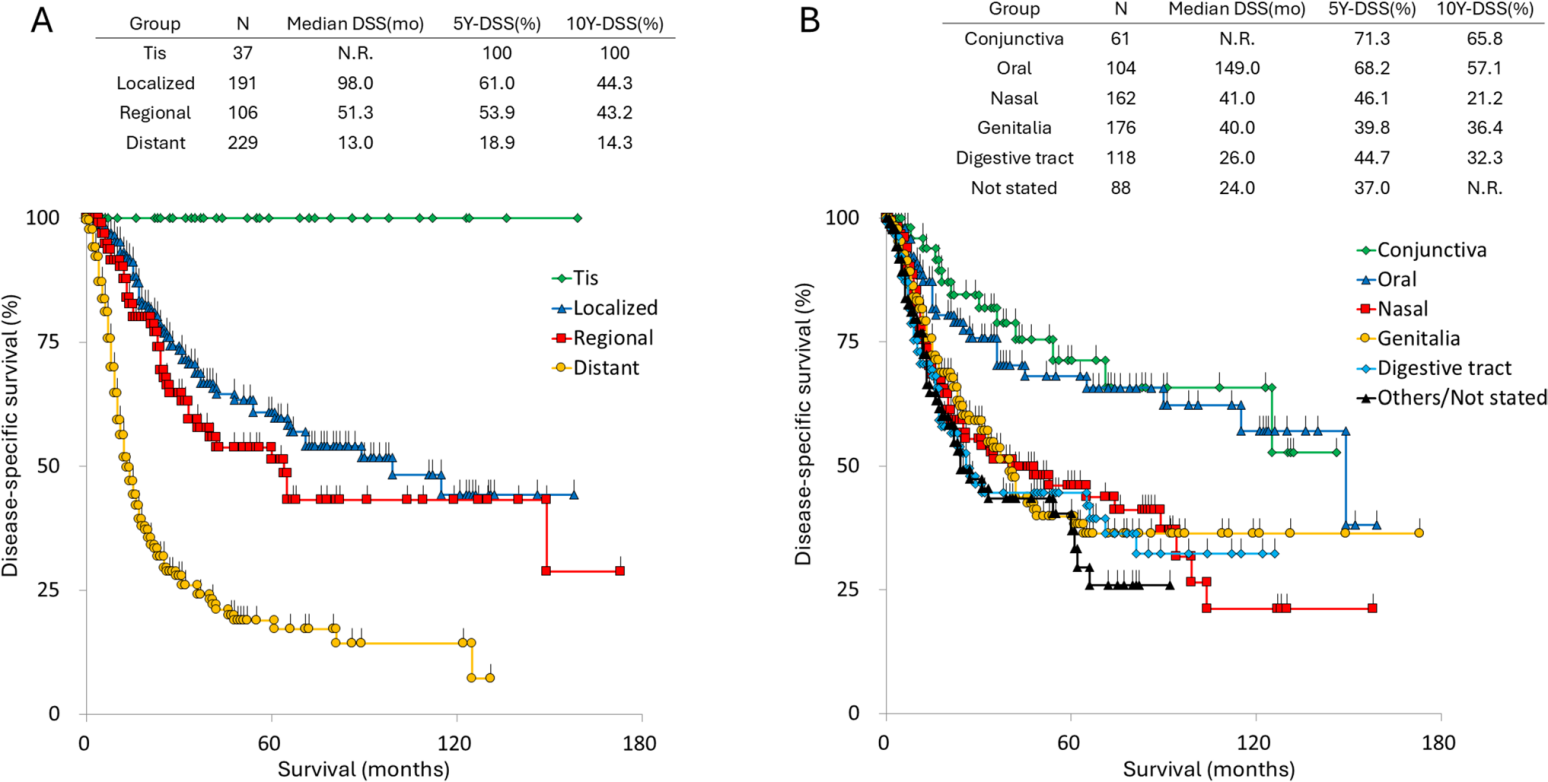
Survival curves of patients with mucosal melanoma. **A** Survival curves stratified by disease stage: Tis, localized, regional, and distant disease. **B** Survival curves stratified by primary tumor sites. 5Y-DSS: 5-year disease-specific survival, 10Y-DSS: 10-year disease-specific survival, N.R.: not reached

A total of 709 patients were diagnosed with mucosal melanoma (MCM). As shown in Table 4, the most common site was the genitalia, 24.8%, followed by nasal cavity at 22.8%; the digestive tract at 16.6%; the oral cavity at 14.7%; conjunctiva at 8.6%; and other/unspecified sites at 12.4%. Survival curves for each category are presented in Fig. 3A. Interestingly, MCM arising in easily noticeable locations (e.g., the conjunctiva and lip/oral cavity) was associated with better survival outcomes than MCM at less easily detectable sites.

**Table 4:** Site and stage of 709 mucosal melanomas

Site	No	%	5Y-DSS	10Y-DSS
Conjunctiva	61	8.6	71.3	65.8
Oral cavity	104	14.7	68.2	57.1
Nasal cavity	162	22.8	46.1	21.2
Digestive tract	118	16.6	44.7	32.3
Genitalia	176	24.8	39.8	36.4
Others/not stated	88	12.4	40.4	N.R

Stage	No	%
Tis	37	5.2
Localized	191	26.9
Regional	106	15.0
Distant	229	32.3
Unclassifiable	146	20.6

*5Y-DSS* 5-year disease-specific survival, *10Y* 10-year, *N.R.* not reached

Since no unified TNM classification system exists for MCM, we broadly categorized the disease into the following groups: Tis, localized disease (no lymphatic or distant involvement), regional disease (nodal involvement), distant disease, and unclassifiable cases. As shown in Table 4, the most common category was distant disease, 32.3%; followed by localized disease including Tis, 32.1%; and regional disease, 15.0%. Survival curves for each category and the 5-year and 10-year DSS rates are presented in Fig. 3.

### Uveal melanoma

**Fig. 4:**
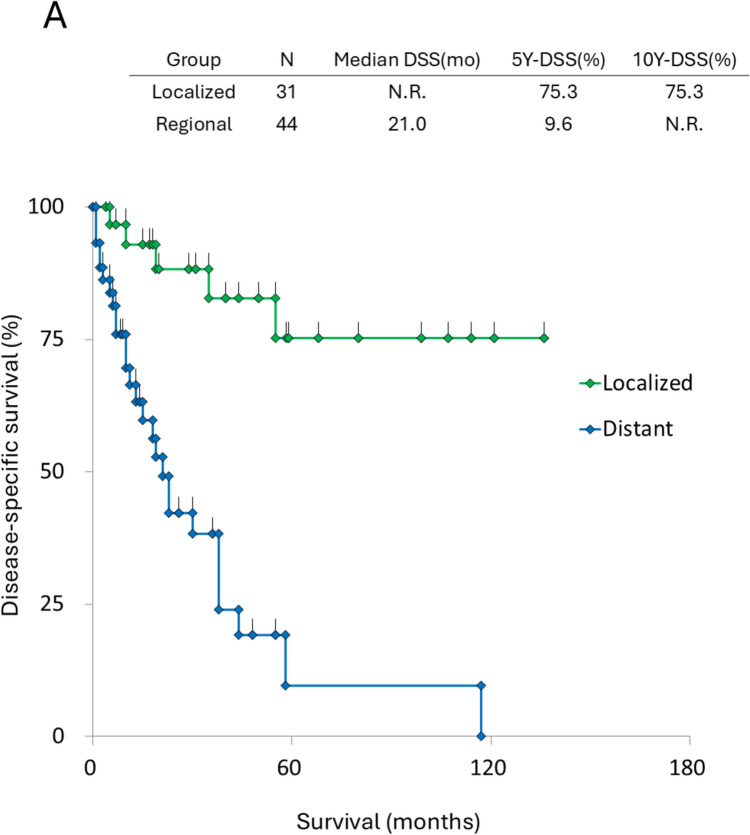
Survival curves of ocular melanoma. **A** Survival curves stratified by disease stage: Tis and distant disease

Regarding uveal melanoma, 75 patients were studied, and 44 of them, 44 (58.7%) presented with distant metastasis at the time of diagnosis. As illustrated in Fig. 4, patients with metastasis at diagnosis experienced significantly worse survival rates (5-year DSS: 9.6% versus 75.3%) compared to those without metastasis (log-rank: *P* < 0.0001).

### *Adjuvant therapy using PD-1 and BRAFi* + *MEKi in patients with cutaneous melanoma*

**Fig. 5:**
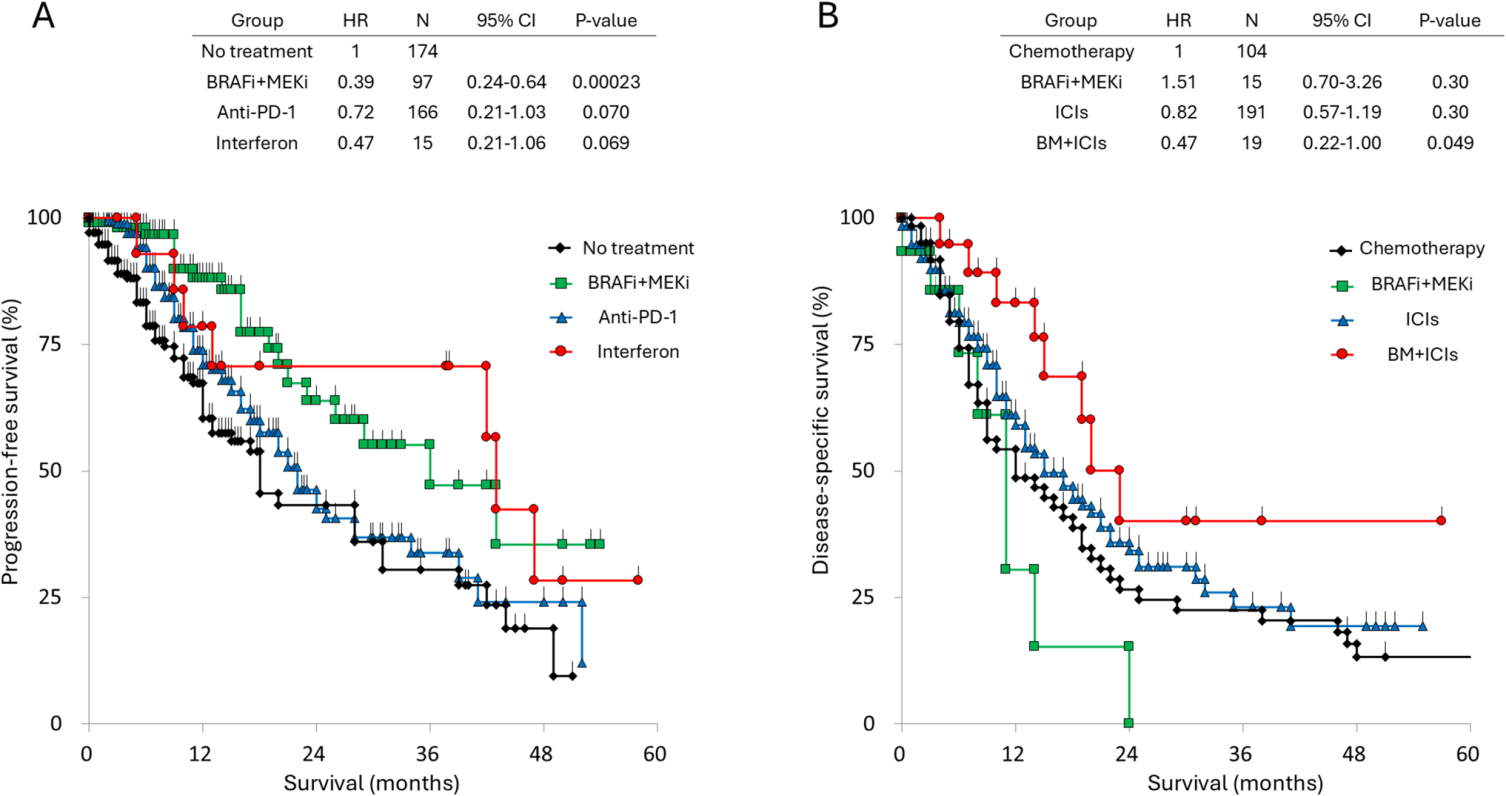
Survival curves of patients with Stage IV disease. **A** Survival curves of patients classified as AJCC 8th Stage III, stratified by adjuvant treatment. **B** Survival curves of patients with Stage IV disease, stratified by treatment received

In Japan, the adjuvant use of anti-PD-1 antibodies and BRAFi + MEKi became available after 2018. Therefore, we analyzed the clinical effects of anti-PD-1 antibodies and BRAFi + MEKi using data from 2018 to 2022. A total of 452 patients with Stage III cutaneous melanoma were identified, of whom 278 (61.5%) received adjuvant therapy. Among these, 166 received anti-PD-1 inhibitors, 97 received BRAFi + MEKi, and 15 received interferon. As shown in Fig. 5A, patients who received BRAFi + MEKi demonstrated significantly better relapse-free survival (RFS) compared to those who did not receive adjuvant therapy (HR 0.39, 95% CI 0.24–0.64, *P* < 0.0005). Although not statistically significant, patients treated with anti-PD-1 antibodies showed a trend toward improved RFS compared to those without adjuvant therapy (HR 0.72, 95% CI 0.21–1.03, *P* = 0.070).

### *Treatment of stage IV disease with ICIs and BRAFi(*+ *MEKi)*

In Japan, both nivolumab and vemurafenib have been approved for use. Of the 808 patients with Stage IV disease, 514 were enrolled after 2014. Since the number of Stage IV patients enrolled after 2014 was only 514, we categorized them into the following groups: patients treated with BRAFi(+ MEKi) but not with ICIs, those treated with ICIs but not BRAFi(+ MEKi), those who received both BRAFi(+ MEKi) and ICIs sequentially (but not concomitantly), those who received chemotherapy without either BRAFi(+ MEKi) or ICIs, and those without available treatment data. Of these, 329 patients had documented records of their received treatments: 191 received immune checkpoint inhibitors (ICIs) but not BRAFi(+ MEKi), 15 received BRAFi(+ MEKi) but not ICIs, 19 received both ICIs and BRAFi(+ MEKi), and 104 received chemotherapy without ICIs or BRAFi + MEKi. DSS curves for each treatment group are presented in Fig. 5B. Although no significant differences were observed, patients treated with ICIs showed improved survival compared to the chemotherapy group (HR 0.82, 95% CI 0.57–1.19, *P* = 0.30). Notably, only patients treated with both ICIs and BRAFi(+ MEKi) demonstrated statistically significant better survival compared to those who received chemotherapy (HR 0.47, 95% CI 0.22–1.00, *P* < 0.05).

## Discussion

In Japan, the incidence of melanoma is reported to be approximately 1–2 cases per 100,000 people per year [1, 2], which is more than ten times lower than the incidence observed in Caucasian populations [1, 9, 10]. Our study shows that the distribution of clinical subtypes differs significantly; ALM is the most common subtype, accounting for more than 40% of cases. Additionally, MCM accounts for nearly 10%, meaning that ALM and MCM together comprise more than half of all melanoma cases in the Japanese population. Consequently, more than half of all melanomas develop in the extremities, while fewer than 30% occur in the head, neck, or trunk.

The clinical subtype not only correlates with the location of the primary tumor but also influences treatment selection. In our study, the percentage of BRAF mutations in each subtype showed that only 8.5% of ALM patients and 4.8% of MCM patients harbored a BRAF mutation, whereas more than 60% of SSM patients carried the mutation. This finding is consistent with the previous study by Sakaizawa K et al.[11]. Consistent with prior studies, less than 30% of Japanese melanoma patients had a BRAF mutation, reflecting the predominance of ALM and MCM subtypes, which are known to have lower mutation rates. Given the low prevalence of BRAF mutations, treatment options for advanced melanoma in Japan are limited, necessitating reliance on ICIs. However, our data showed suboptimal outcomes with ICIs, with no statistically significant improvement in RFS (HR 0.72, 95% CI 0.21–1.03, *P* = 0.070) and DSS (HR 0.82, 95% CI 0.57–1.19, *P* = 0.30).

As shown in Table 2, among patients diagnosed with cutaneous melanoma between 2005 and 2017, 68.4% had localized disease, 26.0% had node-positive disease, and 5.6% had distant metastases. A similar trend was observed in patients diagnosed after 2018 (Table 3), suggesting that the proportion of patients diagnosed at Stage III or Stage IV remained consistent. According to the National Cancer Institute (NCI) website [12], the proportion of patients with localized, regional, and distant disease in United States is reported to be 77%, 10%, and 5%, respectively. Interestingly, while the proportion of distant disease is comparable, the proportion of regional disease is significantly higher in the Japanese population compared to NCI data. The proportion of patients with lymph node metastasis did not differ considerably among clinical subtypes; therefore, we currently have no clear explanation for the high prevalence of lymph node metastasis in the Japanese population.

Regarding survival, the 5-year DSS reported by Balch CM et al. [13] for the AJCC 7th staging system was 99%, 97%, 94%, 87%, and 82% for Stages IA, IB, IIA, IIB, and IIC, respectively. For Stages IIIA, IIIB, and IIIC, the reported 5-year DSS was 78%, 59%, and 40%, respectively. Interestingly, among Japanese cutaneous melanoma patients, the 5-year DSS for Stage IIC was approximately 10% lower than that reported in Western populations, whereas survival rates for other stages were broadly consistent. This discrepancy may reflect the relatively high average tumor thickness of 3.90 mm among Japanese patients, suggesting that locally advanced disease is more common in this population. This finding aligns with prior reports indicating that Asian melanoma patients tend to present with more advanced primary tumors.

As shown in Fig. 3B, MCM patients with conjunctiva and oral cavity involvement exhibited superior survival compared to those with tumors on other sites. Interestingly, there was only a 10% difference in 5-year DSS between localized disease (excluding Tis) and regional disease (Fig. 3A). Since patients with Tis had excellent survival outcomes, early recognition of MCM is crucial for improving prognosis. However, MCM arising in the nasal cavity or digestive tract may be difficult to for patients and clinicians to detect. Therefore, developing new screening methods, such as the early detection of circulating tumor cells, is highly needed to facilitate early diagnosis and improve survival outcomes.

To reduce recurrence or metastasis, anti-PD-1 and BRAFi + MEKi became available for adjuvant use in Stage III melanoma in Japan in 2018. We analyzed the effect of adjuvant therapy on RFS among Stage III patients enrolled after 2018 (Fig. 5A). However, we did not find a statistically significant benefit for anti-PD-1 therapy. In contrast, BRAFi + MEKi demonstrated a statistically significant survival benefit compared to the untreated group. Muto Y et al. [14] reported that anti-PD-1 therapy resulted in inferior survival outcomes compared to BRAFi + MEKi, which is consistent with our findings. Although Jacques et al. [15] showed that adjuvant anti-PD-1 therapy was effective in 139 Stage III ALM patients from 20 centers across 10 countries, this discrepancy may be explained by the fact that ALM, the most common clinical subtype, has been shown to harbor a lower tumor mutational burden [16], which correlates with a reduced response to anti-PD-1 therapy.

Nivolumab was approved in Japan in July 2014, making it the first country to approve this therapy. Following this approval, several other treatment options for advanced melanoma have been introduced, including vemurafenib, pembrolizumab, ipilimumab in combination with nivolumab, dabrafenib plus trametinib, and encorafenib plus binimetinib. Among patients who received treatment for Stage IV disease, only those treated with both ICIs and BRAFi(+ MEKi) demonstrated a statistically significant survival benefit compared to the chemotherapy group (Fig. 5B, *P* < 0.05). While patients treated with ICIs showed better survival than those receiving chemotherapy, the difference was not statistically significant. In contrast, patients treated only with BRAFi(+ MEKi) showed no survival benefit. In clinical practice, most patients who develop resistance to BRAFi(+ MEKi) subsequently receive ICIs [17]. Therefore, those who were treated solely with BRAFi(+ MEKi) may not have had the opportunity to switch to ICIs due to rapid tumor progression.

However, this study has several limitations. First, its retrospective design may introduce selection bias. Second, missing mutation data could affect the accuracy of genetic profiling and treatment response analyses. Third, the follow-up period for the 2018–2022 cohort remains incomplete, limiting long-term survival assessments. To address these limitations, JMS has now been integrated into the Japan Skin Cancer Registry Study (JSCaRS), enabling continuous data collection and long-term follow-up. This transition will facilitate more comprehensive analyses of melanoma treatment outcomes and potentially uncover new prognostic and predictive factors specific to Japanese patients.

## Conclusion

This study highlights the distinct epidemiological and clinical characteristics of Japanese melanoma patients, with a predominance of ALM and MCM and lower BRAF mutation rates. Given the limited efficacy of ICIs in this cohort, optimizing immunotherapy strategies remains a critical challenge. Immediate approaches include the neoadjuvant use of ICIs[18], the approval of relatlimab [19], and combination therapy with other anticancer agents, as demonstrated in other malignancies [20, 21]. Future studies should explore biomarker-driven approaches, such as PD-L1 expression, tumor mutational burden, and gut microbiota profiling, to optimize immunotherapy strategies and improve outcomes for Japanese melanoma patients.
