# Correction to: Melanoma skin cancer statistics derived from 7442 Japanese patients: Japanese melanoma study

**DOI:** 10.1007/s10147-025-02814-1

**Published:** 2025-06-26

**Authors:** Yasuhiro Fujisawa, Shusuke Yoshikawa, Tatsuya Takenouchi, Shoichiro Mori, Jun Asai, Hisashi Uhara, Yuki Ichigosaki, Taku Fujimura, Yoshiyuki Nakamura, Yasuhiro Nakamura, Fumitaka Ohno, Takeshi Fukumoto, Toshiyuki Ozawa, Kenjiro Namikawa, Satoru Sugihara, Toshihiko Hoashi, Takatoshi Shimauchi, Yu Sawada, Hiroaki Iwata, Taku Maeda, Takuya Miyagawa, Yoshitsugu Shibayama, Naohito Hatta, Akiko Kishi, Masashi Ishikawa, Hisao Kawahira, Norito Katoh, Ryuhei Okuyama

**Affiliations:** 1 Department of Dermatology, Ehime University, 454 Shizugawa, Toon, Ehime 791-0204 Japan; 2 Department of Dermatology, Shizuoka Cancer Center, Nagaizumi, Japan; 3 Division of Dermatology, Niigata Cancer Center Hospital, Niigata, Japan; 4 Department of Dermatology, Nagoya University, Nagoya, Japan; 5 Department of Dermatology, Kyoto Prefectural University of Medicine Graduate School of Medical Science, Kyoto, Japan; 6 Department of Dermatology, Sapporo Medical University, Sapporo, Japan; 7 Department of Dermatology and Plastic Surgery, Kumamoto University, Kumamoto, Japan; 8 Department of Dermatology, Tohoku University Graduate School of Medicine, Sendai, Japan; 9 Department of Dermatology, Institute of Medicine, University of Tsukuba, Tsukuba, Japan; 10 Department of Skin Oncology/Dermatology, Saitama Medical University International Medical Center, Hidaka, Japan; 11 Department of Dermatology, Graduate School of Medical Sciences, Kyushu University, Fukuoka, Japan; 12 Division of Dermatology, Department of Internal Related, Graduate School of Medicine, Kobe University, Kobe, Japan; 13 Pharmaco-Physiology & Kinetics Collaborative Research Division, Graduate School of Medicine, Osaka Metropolitan University, Osaka, Japan; 14 Department of Dermatologic Oncology, National Cancer Center Hospital, Tokyo, Japan; 15 Department of Dermatology, Okayama University, Okayama, Japan; 16 Department of Dermatology, Nippon Medical School, Bunkyo, Japan; 17 Department of Dermatology, Hamamatsu University School of Medicine, Hamamatsu, Japan; 18 Department of Dermatology, University of Occupational and Environmental Health, Hakata, Japan; 19 Department of Dermatology, Gifu University Graduate School of Medicine, Gifu, Japan; 20 Department of Plastic and Reconstructive Surgery, Hokkaido University Hospital, Sapporo, Japan; 21 Department of Dermatology, University of Tokyo Graduate School of Medicine, Tokyo, Japan; 22 Department of Dermatology, Faculty of Medicine, Fukuoka University, Fukuoka, Japan; 23 Department of Dermatology, Toyama Prefectural Central Hospital, Toyama, Japan; 24 Department of Dermatology, Toranomon Hospital, Tokyo, Japan; 25 Department of Dermatology, Saitama Cancer Center Hospital, Ina, Japan; 26 Department of Dermatology, Kagoshima University Graduate School of Medical and Dental Sciences, Kagoshima, Japan; 27 Department of Dermatology, Shinshu University, Matsumoto, Japan

**Correction to: International Journal of Clinical Oncology (2025) 30:844–855** 10.1007/s10147-025-02747-9

The article Melanoma skin cancer statistics derived from 7442 Japanese patients: Japanese melanoma study written by Yasuhiro Fujisawa, Shusuke Yoshikawa, Tatsuya Takenouchi, Shoichiro Mori, Jun Asai, Hisashi Uhara, Yuki Ichigosaki, Taku Fujimura, Yoshiyuki Nakamura, Yasuhiro Nakamura, Fumitaka Ohno, Takeshi Fukumoto, Toshiyuki Ozawa, Kenjiro Namikawa, Satoru Sugihara, Toshihiko Hoashi, Takatoshi Shimauchi, Yu Sawada, Hiroaki Iwata, Taku Maeda, Takuya Miyagawa, Yoshitsugu Shibayama, Naohito Hatta, Akiko Kishi, Masashi Ishikawa, Hisao Kawahira, Norito Katoh and Ryuhei Okuyama was originally published under exclusive license to Japan Society of Clinical Oncology, on April 7, 2025 without Open Access. As a result of the subsequent decision to publish the article under the open access model, the article’s copyright notice was changed on 17 June 2025 to © The Author(s) 2025 and the article is now distributed under a Creative Commons Attribution 4.0 International License, which permits use, sharing, adaptation, distribution and reproduction in any medium or format, as long as you give appropriate credit to the original author(s) and the source, provide a link to the Creative Commons licence, and indicate if changes were made.

The images or other third party material in this article are included in the article’s Creative Commons licence, unless indicated otherwise in a credit line to the material. If material is not included in the article’s Creative Commons licence and your intended use is not permitted by statutory regulation or exceeds the permitted use, you will need to obtain permission directly from the copyright holder.

To view a copy of this licence, visit http://creativecommons.org/licenses/by/4.0.

The original article has been updated.

